# Supplementary material for: Bayesian refinement of protein structures and ensembles against SAXS data using molecular dynamics
Source: PLoS Comput Biol. 2017 Oct 18;13(10):e1005800. doi: 10.1371/journal.pcbi.1005800 (PMC5662244; doi:10.1371/journal.pcbi.1005800)
Supplement: S1 Archive — (BZ2) [file pcbi.1005800.s011.tar.bz2 › gromacs_4.62_bayesian_saxs_refi/share/html/online/genconf.html]

genconf


| genconf Main Table of Contents |

**VERSION 4.6  
Sat 19 Jan 2013**

---

### Description

genconf multiplies a given coordinate file by simply stacking them
on top of each other, like a small child playing with wooden blocks.
The program makes a grid of user-defined
proportions (-nbox),
and interspaces the grid point with an extra space -dist.

When option -rot is used the program does not check for overlap
between molecules on grid points. It is recommended to make the box in
the input file at least as big as the coordinates +
van der Waals radius.

If the optional trajectory file is given, conformations are not
generated, but read from this file and translated appropriately to
build the grid.

### Files

| option | filename | type | description |
| --- | --- | --- | --- |
| **-f** | conf.gro | Input | Structure file: gro g96 pdb tpr etc. |
| **-o** | out.gro | Output | Structure file: gro g96 pdb etc. |
| **-trj** | traj.xtc | Input, Opt. | Trajectory: xtc trr trj gro g96 pdb cpt |

### Other options

| option | type | default | description |
| --- | --- | --- | --- |
| **-[no]h** | bool | no | Print help info and quit || **-[no]version** | bool | no | Print version info and quit || **-nice** | int | 0 | Set the nicelevel || **-nbox** | vector | 1 1 1 | Number of boxes || **-dist** | vector | 0 0 0 | Distance between boxes || **-seed** | int | 0 | Random generator seed, if 0 generated from the time || **-[no]rot** | bool | no | Randomly rotate conformations || **-[no]shuffle** | bool | no | Random shuffling of molecules || **-[no]sort** | bool | no | Sort molecules on X coord || **-block** | int | 1 | Divide the box in blocks on this number of cpus || **-nmolat** | int | 3 | Number of atoms per molecule, assumed to start from 0. If you set this wrong, it will screw up your system! || **-maxrot** | vector | 180 180 180 | Maximum random rotation || **-[no]renumber** | bool | yes | Renumber residues |

### Known problems

- The program should allow for random displacement of lattice points.

---

http://www.gromacs.org  
gromacs@gromacs.org
